# Association between subjective well‐being and perception of medical care system among patients with Marfan syndrome: A cross‐sectional study

**DOI:** 10.1002/mgg3.1661

**Published:** 2021-05-05

**Authors:** Tomoko Shimizu, Yasuko Shimizu

**Affiliations:** ^1^ Division of Health Sciences Osaka University Graduate School of Medicine Suita Osaka Japan

**Keywords:** comprehensive health care, intersectoral collaboration, Marfan syndrome, marital status, subjective well‐being

## Abstract

**Background:**

This study investigated the association between subjective well‐being and perception for collaboration among clinical departments of adult Marfan syndrome (MFS) patients.

**Methods:**

We performed a self‐administered questionnaire survey to ask about current medical treatment and support systems and subjective well‐being for 114 patients with MFS aged 18–64 years. It was hypothesized that patients’ perception of collaboration between clinical departments would raise their subjective well‐being. Mean value differences were predicted by a multiple regression analysis model, with supportive medical staff, age, sex, aorta dissection, family history, marriage status, and educational background adjusted.

**Results:**

Patients’ perception of collaboration between clinical departments and being married raised SWLS scores (mean difference for patients’ perception of collaboration versus not = 3.41, 95% CI = 0.28, 6.53, *p* = .03; for married versus single = 5.22, 95% CI = 1.75, 8.69, *p* = .003).

**Conclusion:**

Our results have suggested that it is necessary to maintain and enhance the medical treatment system with the patients for improving the subjective well‐being of MFS patients. In addition, the result indicated the need for intervention to the patients themselves and also their family so that it allows patients to receive physical and emotional support from people close to them.

## Graphical abstract

Patients’ perception of collaboration between clinical departments and being married raise SWLS scores.

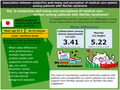

## 1 INTRODUCTION

Marfan syndrome (MFS) is an autosomal dominant hereditary disorder of connective tissues. The cause of MFS was thought to be primarily mutation in the *FBN1* gene (MIM: 134797). The dysfunctional transforming growth factor (TGF‐β) cytokine plays a more critical role in extracellular matrix homeostasis or remodeling (Gonzales, 2009; Neptune et al., 2003). Ghent criteria 1 and 2 have been used for diagnoses since 1996 and 2010, respectively (De Paepe et al., 1996; Loeys et al., 2010). In the revised Ghent criteria for diagnosis of MFS, genetic factors such as a pathological gene mutation and a family history are more important than skeletal findings. MFS occurs in approximately 2–3 per 10000 individuals (Judge & Dietz, 2005). Approximately 25% of cases are caused by de‐novo mutations, have no family history (Judge & Dietz, 2005). The phenotypic expression includes skeletal, pulmonary, ocular, and cardiovascular manifestations, of which dilation and dissection of the aorta are most serious. Cardiovascular complications are the most common causes of early death, and therefore medical management of the cardiovascular system is particularly important. Furthermore, they have various psychosocial problems such as prejudice, bullying, hesitation to marriage or pregnancy, influence on work, etc (Shimizu & Shimizu, 2019).

Some surveys for MFS patients shows that their QOL is lower than that of non‐patients (Rand‐Hendriksen et al., 2010; Rao et al., 2016) while other surveys (Mueller et al., 2016) shows that there isn’t much of a difference in QOL scores between the patients and non‐patients in the younger generation especially. Age, education history, marriage status, income, insurance, job situation, alcohol intake status, the number of surgeries, complications, depression, etc., have been reported as factors to influence the QOL of MFS patients (Goldfinger et al., 2017). Further, it has been reported that QOL of the group which had emergency surgeries was lower than that of the group of elective surgeries (Song et al., 2012), severity was not related to QOL (Goldfinger et al., 2017), and correlation with ascending aorta surgery, use of β blocker, eyesight, and joint mobility were not seen (Glover & Maron, 1998). A study that investigated 117 Norwegian adult MFS patients using life satisfaction level scales (Velvin et al., 2016) reported that the disease had a significant association with severe fatigue, psychological burden, and aorta dissection although it did not with cardiovascular surgery, visual impairment, chronic pain, and working situation. Although united opinions were not obtained, the result showed that their QOL and life satisfaction levels were associated with the presence of aorta lesions and experience in cardiovascular surgeries, psychological burden, job situation, and long‐term influence caused by the disease.

Our precedent study (Shimizu et al., 2017) revealed that adult MFS patients had negative emotions for a disease, while they had an attitude to live valuing their life. They seemed to change the view of life and update the way of feeling their own situation and meaning to the disease experience in their experience of disease itself and treatment. Moreover, the result of this precedent study suggested that it is important to support MFS patients so that they can erase the negative attitude for MFS because they cannot expect to heal. We presumed that MFS patients evaluated their entire life subjectively without being influenced by the disease and severity of the symptoms. Subjective well‐being is also called subjective happiness. It is one of the excellent functions that humans originally have and is own evaluation for own private life (Diener et al., 1999). We assumed that how the person recognizes his/her own life, subjective well‐being in other words would be important when considering care provision for MFS patients. In addition, we focused on subjective well‐being assuming that subjective well‐being, which can hardly be affected by the disease and physical conditions in comparison with HRQOL, is one of the important goals in medical care, including living their lives as who they are. The precedent studies (Velvin et al., 2016; Stanišić et al., 2018) verified internal factors of patients and subjective well‐being, while its relationships with external factors such as medical environments surrounding the patients were not examined. We focused collaboration situation among clinical departments as a medical environment of MFS patients who needed to consult plural clinical departments due to multiple symptoms.

We hypothesized that the patients’ subjective well‐being rises when they recognize collaboration among the clinical department. This study aimed to examine is there are associations between subjective well‐being and perception for collaboration among clinical departments of adult MFS patients. We believe that the result of this study will enable us to consider effective support to improve the subjective well‐being of MFS patients.

## 2 METHODS

### 2.1 Participants

Participants in this study were individuals aged 18–64 years who self‐reported being diagnosed with Marfan syndrome (MFS) and were able to self‐administer the questionnaire. Participants were recruited through the Marfan Network Japan and the Japan Marfan Association in Japan.

### 2.2 Procedure

The authors conducted a cross‐sectional questionnaire survey from July to December 2019. Questionnaire sheets were distributed by mail to each adult with MFS and sent back in a reply envelope with their answers in them. Two hundred and seventy persons were asked to complete the survey and 114 of them returned the questionnaire (response rate was 42.2%). Since the frequency of occurrence was estimated to be approximately 2–3 per 10,000 people (Judge & Dietz, 2005), the estimated number of patients with MFS in our country was estimated to be approximately 25,000–38,000.

### 2.3 Measures

A study‐specific questionnaire included questions about demographic characteristics and medical care systems, and the Satisfaction with life Scale (SWLS) was used. The questionnaire design was based on the past literature and our clinical experience. We also requested a Cognitive test from a colleague in the laboratory to ensure that the questions were communicated and that there were no questions that were difficult to answer. In addition, to ensure validity, we sought the advice of a physician in the Department of Genetics who is familiar with the treatment of patients with MFS.

1. Demographic factors
  Sex, age, age at diagnosis, marital status, family history (yes/no), educational level (</ ≥13 years), job (yes/no), annual income, medical expenses, physical disability certificate (yes/no), emergency cardiovascular surgery (yes/no), diagnostic features.
2. Perceptions of the Medical care/support system
  Management of individuals with MFS requires holistic care (Gonzales, 2009; Yip & Sawatzky, 2014). Multidisciplinary health care for patients with MFS is effective for improving physical fitness and psychological well‐being (Benninghoven et al., 2017). From the above, we assumed that medical treatment and support system influenced the patients’ subjective well‐being. In this study, we investigated the perception of the collaboration among clinical departments in which the patients were consulting and the presence of medical persons whom they could consult with as perception of medical treatment and support system.
3. The Satisfaction with life Scale (SWLS)
  The Satisfaction with life scale (SWLS; Diener et al., 1985) is widely used for measuring subjective well‐being. SWLS is a valid and reliable measure used for patients with MFS overseas, and a Japanese version has also been prepared. SWLS is a measure of subjective well‐being self‐assessment consisting of five questions, each of which is to be answered based on a Likert scale ranging from “strongly disagree” to “strongly agree” with corresponding scores ranging from 1 to 7. The total sum score ranged from 5 to 35. Total scores are categorized as: Extremely satisfied (31–35), Satisfied (26–29), Slightly satisfied (21–25), Neutral (20), Slightly dissatisfied (15–19), Dissatisfied (10–14), and Extremely dissatisfied (5–9) (Pavot & Diener, 2008).

### 2.4 Data analysis

First, descriptive statistics analysis was performed to determine the mean, standard deviation, frequency distribution, and percentage of each question item. The internal consistency of the SWLS scores was determined by the Cronbach alpha coefficient. In order to investigate the association between levels of SWLS scores and the presence of collaboration among clinical departments, we conducted multiple linear regression analyses and obtained mean differences and 95% confidence intervals (CI). Multivariable analysis was adjusted for the presence of supportive medical staff, age, sex, aorta dissection, family history, marital status, and education level. We defined income, work and psychological burden as intermediate variables in our analytical model, because they were influencing factors to SWLS. In addition, for symptoms other than aortic dissection, a *p* value of .3 or higher in the single regression analysis was regarded not significant and it was not included as an independent variable. Potential confounder selection was based on previous studies and multicollinearity. Data were analyzed by the statistical analysis software R for Windows (ver3.6.1), and the following statistical processing was performed. The significance level was set at *p* < .05 for all the statistical tests.

### 2.5 Ethical considerations

We explained to the subjects using a letter of intent that survey participation was not compulsory, they had the right to withdraw from the participation at any time, non‐consent for survey participation entailed no disadvantages, and personally, identifiable information would not be disclosed to third parties. They expressed their willingness to participate in the study by checking in the box in the questionnaire and we obtained their consent properly. This study was carried out upon approval by the Ethics Committee of Osaka University Hospital (approval number: 18517‐2).

## 3 RESULTS

### 3.1 Participant’ characteristics

Participant demographic information is reported in Table 1. The participants’ mean age was 45.4 years (SD = 12.4) and 56.1% of them were women. The mean age at diagnosis was 22.4 years (SD =14.6 range: 0–53). Age at diagnosis ≤20 was 50.8% and 66 (57.9%) of the participants had a least one relative affected with Marfan syndrome. Approx. 60% of the respondents were married. Moreover, 42.1% of respondents had children and 45.6% answered they did not. Approx. 70% reported they had completed a formal education of 13 years or more and 63.8% of the participants earned less than 4 million yen annually. Many participants had cardiovascular complications, with valvular heart disease at 43.9%, aortic dissection at 39.5%, and aortic aneurysm at 38.6%, and 60.5% had a certificate of the physically disabled.

**TABLE 1:** Demographic and clinical characteristics for study participants (n=114)

	Mean (SD)		Range
Age	45.4 (12.4)		[18–64]
Age at diagnosis	22.4 (14.6)		[0–53]
Monthly medical cost (yen)	3110 (3678.7)		[0–20000]

		n (%)	
Sex			
Man		50 (43.9)	
Women		64 (56.1)	
Family history			
No		47 (41.2)	
Yes		66 (57.9)	
Unknown		1 (0.9)	
Marital status			
Married		67 (58.8)	
Single (separation by death included)		47 (41.2)	
Presence of child			
No		52 (45.6)	
Yes		48 (42.1)	
No answer		14 (12.3)	
Presence of housemate			
No		19 (16.7)	
Yes		95 (83.3)	
Final academic background			
<13years		32 (28.1)	
≥13 years		81 (71.1)	
Don't want to answer		1 (0.9)	
Job			
No/full‐time housewife or househusband		32 (28.1)	
Yes		95 (83.3)	
Annual income(n=94)			
Less than 4 million yen		60 (63.8)	
More than 4 million yen		26 (27.7)	
Don't want to answer		8 (8.5)	
Experience of emergency cardiovascular surgery			
No		54 (47.4)	
Yes		60 (52.6)	
Diagnostic features			
Aorta dissection		45 (39.5)	
Aortic aneurysm		44 (38.6)	
Valvular disease		50 (43.9)	
Skeletal issues		32 (28.1)	
Lens dislocation		40 (35.1)	
Certificate of the physically disabled			
No		39 (34.2)	
Yes		69 (60.5)	
No answer		6 (5.3)	

### 3.2 Perceptions of the medical care/support system

The subjects who visited more than two clinical departments were asked about their perceptions of the status of collaboration among departments. The subjects who visited more than two clinical departments to have medical care were asked if there was a collaboration between the departments. Seven subjects did not need to answer this question since they received medical care at only one clinical department. Furthermore, 14 subjects did not respond, 35 (30.7%) of the respondents were aware that there was a collaboration among clinical departments, and 83 (72.8%) answered that there was medical staff available for consultation (Table 2).

**TABLE 2:** Perceptions of Medical care/support system (n=114)

	n (%)
Collaboration among clinical departments	
No	58 (50.9)
Yes	35 (30.7)
No answer	14 (12.3)
N/A*	7 (6.1)
Supportive medical staff	
No	23 (20.2)
Yes	83 (72.8)
No answer	83 (72.8)

* Respondents who consulted only one clinical department were categorized in N/A.

### 3.3 SWLS score in patients with MFS

The internal consistency of the SWLS was evidenced by an alpha coefficient of 0.81. The inter‐item correlation was 0.70. The total mean sum SWLS score was 20.0 (SD7.1). More than half scored 20 or more, indicating that they were satisfied with their life. The lowest score was found for the SWLS item “If I could live my life over again, I would change almost nothing about my life,” and the highest score was “So far I have gotten the important things I want in my life” (Table 3).

**TABLE 3:** Satisfaction with Life Scale, total and item scores (n=101)

	Mean (SD)
Total score	20.0 (7.1)
Score of each item	
1. So far I have gotten the important things I want in my life	5.0 (1.7)
2. I am satisfied with my life	4.3 (1.7)
3. The condition of my life are excellent	4.1 (1.8)
4. In most ways my life is close to my ideal	3.7 (1.7)
5. If I could live my life over, I would change almost nothing	3.0 (1.8)

Note

The items are ranked by descending mean scores.

### 3.4 Association between SWLS and perceptions of medical care/support systems

We investigated the association between patients’ perception of collaboration between clinical departments and SWLS (Table 4). With the presence of supportive medical staff, age, sex, aorta dissection, family history, marriage status, and education level adjusted, the multivariable linear regression analysis revealed a significant association between patients’ perception of collaboration between clinical departments and SWLS (mean difference for patients’ perception of collaboration versus not = 3.41, 95% CI = 0.28, 6.53, *p* = .03). Being married had a significantly higher SWLS score than not being married did(mean difference = 5.22, 95% CI = 1.75, 8.69, *p* = 0.003).

**TABLE 4:** Multivariable linear regression analysis to assess the association between SWLS and perceptions of medical care/support systems (n=85)

	Mean (95% CI)	*p*‐value
Collabration among clinical departments	3.41 (0.28, 6.53)	.03*
Supportive medical staff	2.22 (−1.29, 5.73)	.21
Age	−0.02 (−0.19, 0.14)	.78
Male (vs female)	2.51 (−0.66, 5.67)	.12
Aorta dissection	−0.87 (−4.01, 2.27)	.58
Family history	0.13 (−3.21, 3.48)	.94
Married	5.22 (1.75, 8.69)	.003*
Education level (≥13)	1.67 (−1.76, 5.10)	.34

* *p* < .05.

## 4 DISCUSSION

This is the first study to determine the association between the subjective well‐being of Japanese patients with Marfan syndrome (MFS) and their perceptions of the medical system.

The total SWLS scores of the MFS patients, who were subjects for this study were similar to those reported in the precedent study (Velvin et al., (2016) conducted in Norway. Comparison of scores for each item revealed that the scores for the items "So far I have gotten the important things I want in my life" and "If I could live my life over, I would change almost nothing" were lower by 0.3–0.5 points than those reported in the precedent study. Japanese MFS patients seem to show negative attitudes toward the life they have spent so far. However, their scores do not differ very much and therefore it was influenced by the difference in ideas and interpretation for the happiness of MFS patients in foreign countries and Japan between the two items.

### 4.1 Patients about SWLS and medical treatment system/support system and their relationship with other factors

The result of the multiple regression analysis has indicated that the perception of collaboration among clinical departments exerts a positive influence on SWLS. Collaboration among clinical departments, multifaceted medical treatment, and comprehensive and continuous medical treatment systems are needed for MFS patients (Gonzales, 2009; Kodolitsch et al., 2016). It has not been reported that the medical environment surrounding a patient influences the patient's QOL and subjective well‐being. The results of this study suggested that the patients’ perception of the medical provision system allowed them to feel satisfied with the contents of medical treatment and nursing and brings a positive influence on their subjective well‐being. However, this study verifies relationships between SWLS and patients’ perception of collaboration among clinical departments and actual collaboration situations have not been clarified. Further, the possibility that it may differ from the collaboration among clinical departments which medical persons think about cannot be ignored.

It has been reported that external regulation of locus of control and life satisfaction level has positive correlations (Stanišić et al., 2018), and the possibility that MFS patients ask medical workers and their families for solutions to the health problem. It has also been reported that they depend on support and assistance from medical professionals and their family to deal with everyday problems that induce disease and stress (Fusar‐Poli et al., 2008), and therefore support from medical persons and their family is highly important for MFS patients. Marriage situations are also an affecter for subjective well‐being, and therefore we inferred that support from medical persons and patient's family improves subjective well‐being. The existence of a spouse enhances subjective well‐being (Diener et al., 2000). Moreover, it has also been reported that subjective happiness degrees are high because a person makes intentional efforts by marriage (Lyubomirsky & Sheldon, 2005). It has been suggested that emotional support from people close to the patients and activities by intentional efforts in married life are important to improve the subjective well‐being of MFS patients.

SWLS has been related to income, work, and psychosocial functioning (Lyubomirsky & Sheldon, 2005). Income, work, and psychological burden were positioned as mediator variables in the multiple regression analysis model of increasing SWLS in this study. Those who perceived collaboration among departments were likely to have higher SWLS because they were more satisfied with their economic and employment status and psychosocial functioning because the quality of treatment and care was better than those who did not perceive it. Future research is needed to identify differences in the content and quality of support according to collaboration between departments, and to investigate the relationship between these differences and economic status, employment status, and psychological burden. Furthermore, the relationship between these factors and subjective well‐being needs to be examined and effective support needs to be considered.

It is notable that medical staff available for consultation was not shown as an affecting factor for subjective well‐being. Although 70% of the patients answered that they had medical staff available for consultation, it did not exert a positive influence on subjective well‐being. Our precedent study (Shimizu et al., 2017) revealed that adult MFS patients closed their eyes to the disease to forget the fear of sudden death and their appearance different from others had been their complex for a long time. It was guessed that even if the patients could consult about the treatment, they kept feeling the difficulty in their life and complicated worries in themselves, and therefore they did not have a chance to have appropriate advice and education for such difficulty and worries from medical persons. They could not evaluate their own life positively due to such a situation, we presumed. Further, it was also influenced the medical treatment system. MFS is a rare disease, and medical institutions that have the capacity of corresponding to it are limited in Japan. Even such special institutions have difficulty in responding to various problems of each patient in a short consultation time. Therefore, even if there are medical persons whom the patients can consult, they do not receive appropriate advice and psychological support for the complicated psychological problems, which seems to be related to the above.

Aorta dissection was an affecting factor for subjective well‐being in the precedent study (Velvin et al., 2016), but similar results were not obtained in this study. It has been suggested that approx. 40% of the feeling of happiness of humans is possibly prescribed by activities performed in daily life, not by genetic factors and social environment (Lyubomirsky & Sheldon, 2005). The subjects in this study may not necessarily have negative feelings about aortic dissection, but may have found meaning in living with MFS while living with aortic dissection. Moreover, the control of aorta dissection in the subjects of this study might have been more satisfactory compared with those of the precedent study (Velvin et al., 2016).

### 4.2 Implication clinical work

Results of this study have revealed that patients’ perception of collaboration between clinical departments improves the subjective well‐being of the patients, suggesting the need of arranging and enhancing the medical treatment system. It has been indicated that approaches based on collaboration among plural professionals and follow‐up that continues through their life are important in the medical management of MFS patients (Gonzales, 2009) although the medical environment that patients expect has not been clarified. It is important for medical persons to maintain medical treatment systems with patients, while focusing on the ideal collaboration among clinical departments that the patients think and the medical treatment system they wish, we presume. In particular, in the current medical treatment situation in Japan that medical institutions that can respond to MFS are limited and they have difficulty in grasping the entire situation of each patient, provision of team medical service by plural professional, and maintenance of a comprehensive medical treatment system are a clinically important action.

It has also been suggested that emotional support from people close to the patients also improves the subjective well‐being of the MFS patients. Medical persons dealing with MFS patients need to coordinate their relationships with the patients and people around them such as their families and give special consideration to it so that the patients can receive support from people close to them. Several interview surveys for several patients revealed that MFS patients feel hesitation to marriage and pregnancy, inferiority complex to their family and children, apology, etc (Connors et al., 2015; Van Tongerloo et al., 1998). Since patients and their families come across various decision‐making scenes in making a future plan such as marriage or pregnancy, it is necessary to present various options to patients and their families by cross‐department collaboration. Moreover, it is difficult for single persons and people living alone to receive support from people close to them, and that leads to discomforts and difficulty in living with the disease possibly, we presume. It is needed to explore the directionality of the support, considering the situation in which the patients can hardly receive support from people close to them, as well as substantial support to them.

## 5 LIMITATIONS AND FUTURE RESEARCH ISSUES

The generalizability of the results of this study is a concern for the following issues. First, the subjects were limited to members of the self‐help group. Results might have been differed by background and sense of values of the Marfan syndrome (MFS) patients who do not belong to the self‐help group, and therefore further investigation is needed in the future. Second, the study included those who self‐reported having been diagnosed with MFS and did not prove that they had received a medical diagnosis. Third, the response rate was approximately 40%, which may have resulted in non‐response errors, such as opinions of those who had not responded to the survey, which did not reflect opinions of those who had not responded. Based on the estimated incidence of Marfan syndrome, the participating population in this study is the population of our country's MFS it was estimated to be about 0.3%–0.5% of the affected population. A weakness of this study is that the investigation of the medical care and support system is limited to patients’ perceptions of the status of collaboration among departments and the availability of medical professionals they can consult. The lack of data on the content and quality of collaboration among medical departments is a weakness of this study. Future studies need to examine how the content and quality of inter‐departmental collaboration affect patients’ subjective well‐being. It is also necessary to design effective multidisciplinary interventions to improve patients’ subjective well‐being. Finally, since this is a cross‐sectional study, we cannot make a definitive statement about the causal relationship between subjective well‐being and perceptions of collaboration among departments. In the future, a prospective design should be applied to the study on the construction and maintenance of the medical care system that patients wish to have and to examine whether it contributes to subjective well‐being or not.

## 6 CONCLUSIONS

In this study, patients’ perception of collaboration between clinical departments and being married exerted a positive influence on the subjective well‐being of Marfan syndrome patients. The need of maintaining medical treatment systems with patients and consideration by which patients can receive support from familiar people such as families has been suggested. It is needed to examine effective medical systems and assistance for improving subjective well‐being in the future.

## 7 ACKNOWLEDGMENTS

We express our sincere appreciation to the members of the Japan Marfan Association and Marfan Network Japan for their understanding of the purpose of this research and for their cooperation in the survey. We gratefully thank Dr. Hiroko Morisaki at Sakakibara Heart Institute for her assistance in data collection. This study was supported by a Grant From Japan Cardiovascular Research Foundation.

## CONFLICT OF INTEREST

All authors have no conflict of interest.

## Data Availability

The data that support the findings of this study are available on request from the corresponding author. The data are not publicly available due to privacy or ethical restrictions.
